# Components of *NOTCH* Signaling for Uterine Cancer Patients' Prognosis

**DOI:** 10.1155/2022/8199306

**Published:** 2022-01-30

**Authors:** Nadezda Lachej, Daiva Dabkeviciene, Julija Simiene, Rasa Sabaliauskaite, Violeta Jonusiene, Vytautas Brasiunas, Ausra Sasnauskiene, Ieva Vaicekauskaite, Birute Brasiuniene, Daiva Kanopiene, Kestutis Suziedelis, Janina Didziapetriene

**Affiliations:** ^1^Clinic of Internal Diseases, Family Medicine and Oncology, Institute of Clinical Medicine, Faculty of Medicine, Vilnius University, LT-10257 Vilnius, Lithuania; ^2^National Cancer Institute, LT-08660 Vilnius, Lithuania; ^3^Institute of Biosciences, Life Sciences Center, Vilnius University, LT-08412 Vilnius, Lithuania

## Abstract

New molecular biomarkers that could have an independent prognostic value in endometrial cancer are currently under investigation. Recently, it was suggested that genetic changes in the Notch signaling pathway could be associated with the development of endometrial carcinoma. This study aimed to determine the expression of the Notch signaling pathway components in tumour and adjacent normal uterine tissue and to evaluate their importance for the survival of uterine cancer patients. The present study was performed on uterine body samples collected from 109 patients and paired adjacent noncancerous endometrial tissue samples. Kaplan–Meier curves and Cox regression were used for survival analyses. Expression alterations of *NOTCH2*, *NOTCH3*, *NOTCH4*, *JAG2*, and *HES1* were evaluated as independent and significant prognostic factors for uterine cancer patients.

## 1. Introduction

Uterine cancer is the sixth most common cancer among women worldwide, counting about 417 000 new cases and 97 000 deaths in 2020 [1]. It is an actual problem in Lithuania since approximately 600–700 cases of uterine cancer are diagnosed in Lithuania every year. It is the third most common malignancy in women after skin (except melanoma) and breast cancer and leading cancer among gynecological cancers in Lithuania [2]. Incidence rates of uterine cancer are increasing globally, primarily because of increased obesity, a significant risk factor for this disease. Other risk factors include growing life expectancy, reduced fertility, and hormone replacement therapy, especially without progestin [3]. The stage of the disease determines treatment options and has a strong influence on patient survival rates. Among women diagnosed at early stages, the 5-year survival rate is almost 82% [4]. In the diagnosis of distant metastases, the 5-year overall survival rate for advanced-stage uterine cancer is only about 25% [5].

The individualized treatment of uterine body cancer is usually based on classical clinical-pathological characteristics such as histological subtype, stage of the disease, myometrial invasion, lymphovascular invasion, and tumour differentiation grade [6]. Malignancies of the body of the uterus can be broadly classified into epithelial malignancies (endometrial carcinomas), mesenchymal malignancies (uterine sarcomas), mixed epithelial and mesenchymal malignancies of the uterus (carcinosarcomas, adenosarcomas, and carcinofibromas), and trophoblastic malignancies [7].

The tumour subtype is an important prognostic factor for the disease outcome, whereas nonendometrioid tumours are of poorer prognosis than the endometrioid subtype [8]. The stage of uterine cancer is associated with increasingly worse survival for the higher stages. Moreover, the deep myometrial invasion, lymphovascular space invasion, and poorly differentiated tumours are factors indicating an increased risk of recurrence and metastasis [6].

Nowadays, classical clinical-pathological characteristics seem insufficient to avoid adjuvant therapy and associated toxicity in patients with a favorable prognosis and, on the other hand, to consider additional treatment modalities to improve survival outcomes in patients with a high risk of a relapse. New molecular biomarkers that could have an independent prognostic value in endometrial cancer are currently under investigation [9].

Genetic changes in the Notch signaling pathway could be associated with the development of endometrial carcinoma [10–12]. Notch signaling is mediated by four Notch receptors (NOTCH1–4) and five transmembrane ligands (jagged 1 and jagged 2 (JAG1 and JAG2), delta-like 1 (DLL1), DLL3, and DLL4), which are called “canonical” ligands [13]. Notch receptors are single-pass transmembrane proteins with two distinct domains: an extracellular ligand-binding domain and an intracellular mediating the signal transduction [14]. After Notch receptor activation, the receptor is cleaved, and the intracellular fragment transposes to the nucleus of the cell, where it regulates the expression of transcription factors, such as the hairy enhancer of split (*HES*) and *Hes*-related (*HEY*) [13, 15]. Notch signaling pathway can regulate other target genes controlled by mTORC2, PI3K, TGF-*β*, NF*κ*B, and HIF1*α* pathways in the nucleus and/or cytoplasm [16]. In the past few decades, due to its functions, the Notch signaling pathway has been considered as a novel therapeutic target [17]. In cancerous tissues, Notch signaling can show tumour suppressive or oncogenic abilities. Aberrant expression of Notch signaling genes and their targets may be associated with cell differentiation, proliferation, tumorigenesis, metastasis formation, and epithelial-mesenchymal transition [18]. Despite increasing evidence indicating the crucial roles of Notch signaling genes in uterine cancer, the clinical significance remains unclear.

Our study aimed to determine the expression of the Notch signaling pathway components in tumour and adjacent normal uterine tissue and to evaluate their importance for the survival of uterine cancer patients. In this article, only results of the association of investigated Notch pathway components and survival of uterine cancer patients are presented.

## 2. Materials and Methods

### 2.1. Patients

The present study was performed on uterine body samples, collected from 109 patients with stages I–IV of uterine body cancer, who underwent surgery during the period 2010–2016 in National Cancer Institute (Vilnius, Lithuania). The censorship date is June 2020. All tumour samples had a paired control sample—adjacent noncancerous endometrial tissue (determined by histopathologists). Before carrying out the study, permission was obtained from the Vilnius Regional Biomedical Research Ethics Committee (protocol no. I-2010-1, issue no. 158200-05-180-43). All samples were collected with the patients' written consent to participate in the study.

All the patients in the study underwent surgical treatment: removal of the uterus (hysterectomy), including adnexa of the uterus, and pelvic and para-aortic lymphadenectomy according to indications. The mean age of the women included in the study was 65.2 ± 9.2 years (range 43−81). The majority of patients were 60−69 years old and 70−79 years old—33.9% of each group (*n* = 37); the age group 50−59—23.9% (*n* = 26). The smallest proportion of subjects were women aged 40−49 (3.7%, *n* = 4) and 80−89 years (4.6%, *n* = 5). Clinical-pathological characteristics of patients are presented in Table 1.

Depending on the stage of the disease and the degree of tumour differentiation, some patients (*n* = 56; 51.4%) underwent postoperative adjuvant radiotherapy according to the National Cancer Institute standards of treatment: some with vaginal brachytherapy (*n* = 28), others with combined radiation therapy, i.e., pelvic external-beam radiation therapy along with vaginal brachytherapy (*n* = 28). A single dose (SD) of pelvic external-beam radiation therapy was administered—1.8–2.0 Gy in 23–28 fractions five days per week, total dose (TD) 46.0–50.4 Gy. Vaginal (intracavitary) brachytherapy was applied once a week, with 5 Gy (SD) at 0.5 cm depth in the vaginal wall during each brachytherapy procedure, using an iridium-192 source. There were three procedures combined with external-beam radiation therapy (TD, 15 Gy) and four procedures with brachytherapy alone (TD, 20 Gy).

Chemotherapy with cisplatin (50 mg/m^2^) or carboplatin dose of AUC 5 (area under the curve) in combination with doxorubicin (50 mg/m^2^) was administered to 16 (14.7%) patients: five postoperative patients received palliative chemotherapy alone for distant metastases, while the remaining patients underwent chemotherapy before or after radiation therapy.

### 2.2. Sample Collection and Gene Expression Technologies

Samples of normal and pathological uterine tissues were frozen in liquid nitrogen and stored at −80°C temperature. The RNA extraction, copy DNA synthesis, and reverse transcription quantitative PCR were performed following the methodology reported in our previous studies [19, 20].

### 2.3. Statistical Analysis of the Data

A sample size of 92 patients was evaluated as efficient to reach 80% power of the survival test with a 30% difference between survival rates and significance level 0.05. Kaplan–Meier curves and Cox regression were used for survival analyses. Age, FIGO stage, histologic type of tumour, and tumour differentiation grade were considered as factors having a valuable influence on the outcome. Consequently, alterations of gene expression were adjusted for the same age and clinical-pathological characteristics in multivariate Cox regression using the enter method. The cutoff for gene expression values was optimized, evaluating the most significant split between survival curves by the hazard ratio (HR) with 95% confidence intervals. *Cutoff Finder* is an available web application that can be accessed via the Internet (http://molpath.charite.de/cutoff) [21]. Associations between categorical variables were evaluated by using a two-sided Chi-square test or Fisher's exact test, as appropriate. When the *P* value was less than 0.05, the differences were considered statistically significant. *SigmaPlot 13*.*0* and *Statistica Basic Academic 13* were used for data analysis.

## 3. Results

### 3.1. Uterine Patient's Overall Survival Rate Depending on Clinical-Pathological Characteristics

**Figure 1:**
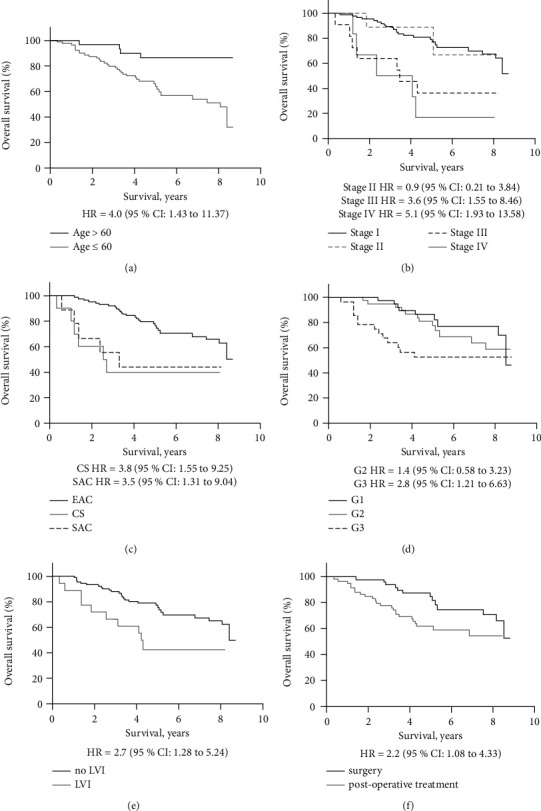
Overall survival rates of uterine cancer patients depending on clinical-pathological characteristics. Survival rates were dependent on as follows: age (a); the stage of the disease (b); the histologic type of tumour: EAC, endometrioid adenocarcinoma; CS, carcinosarcoma; and SAC, serous adenocarcinoma (c); the degree of tumour differentiation (G1, well-differentiated tumour; G2, moderately differentiated tumour; and G3, poorly differentiated tumour) (d); lymphovascular invasion (LVI) (e); treatment (f).

The analysis of uterine patient overall survival depending on clinical-pathological characteristics data showed that females younger than age 60 with uterine cancer had a significantly longer survival rate than older patients (*p* = 0.008; Figure 1(a)). As expected, the disease stage significantly influenced the survival rate (*p* = 0.001; Figure 1(b)). Patients with histologically confirmed endometrioid adenocarcinoma had a better survival rate than those with other histologic forms—carcinosarcoma and serous adenocarcinoma (*p* = 0.002; Figure 1(c)). The degree of tumour differentiation was a significant determinant for survival, as the lowest degree of tumour differentiation (G3) was characterized by low survival rates (*p* = 0.04; Figure 1(d)). Significantly better survival rates were evaluated for patients without lymphovascular invasion (*p* = 0.009; Figure 1(e)). However, the myometrial invasion had no significant effect on patient survival rates (*p* = 0.83). Our results revealed that patients who underwent surgery alone had significantly better survival rates than those receiving postoperative radiation therapy or/and chemotherapy (*p* = 0.03; Figure 1(f)).

### 3.2. Uterine Patient's Survival Rate Depending on the Expression of the Notch Signaling Pathway Components

**Figure 2:**
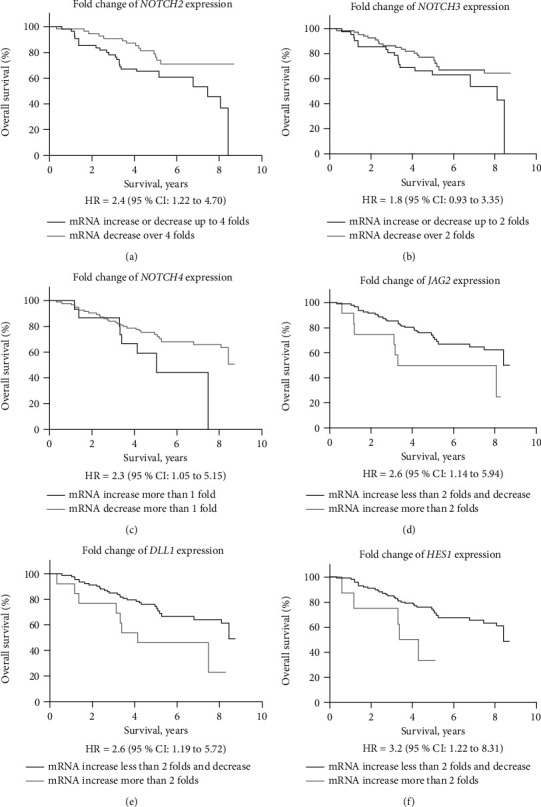
Survival rates of uterine cancer patients depending on the fold change of the *NOTCH* signaling pathway gene expression. *NOTCH2* (a); *NOTCH3* (b); *NOTCH4* (c); *JAG2* (d); *DLL1* (e); *HES1* (f).

The study evaluated the potential influence of the investigated Notch signaling pathway components on survival rates in patients with uterine cancer (Figure 2). The analysis of the obtained data showed a statistically significant association between the survival rate of uterine cancer patients and the expression of *NOTCH2* gene (Figure 2(a)). In the case of higher *NOTCH2* gene expression, the disease prognosis is worse (*p* = 0.01). Decreased *NOTCH3* gene expression had shown a trend toward better overall survival rates (*p* = 0.08) (Figure 2(b)). Changes in *NOTCH4* expression in tumour tissue were significantly associated with patient survival rates (Figure 2(c)). Patients with higher *NOTCH4* expression levels had worse survival rate levels (*p* = 0.03). *JAG2*, *DLL1*, and *HES1* gene expression changes lower than two folds were associated with better overall survival rate (*p* = 0.02 for each gene) (Figure 2(d)–2(f)).

The correlation between subgroups of the Notch signaling pathway gene expression and clinical-pathological characteristics of the patients were evaluated and are presented in Supplementary Materials. Changes in *NOTCH2* expression were significantly associated with tumour differentiation grade (*p* = 0.03, Table S1) while *HES1* expression—with tumour histological type (*p* = 0.007, Table S2).

### 3.3. Multivariate Analysis of Patients with Uterine Cancer

Multivariate Cox regression analysis was performed to adjust gene expression to age and clinical-pathological characteristics, including stage, histology, and tumour differentiation degree. Multivariate analysis identified expression alterations of *NOTCH3* and *NOTCH4*, as a significantly independent prognostic factor for overall survival in patients with uterine cancer. Expression alterations of *NOTCH2*, *JAG2*, and *HES1* were very significantly associated with more prolonged overall survival (Table 2). In addition, age (*p* < 0.01) and disease stage (*p* < 0.001) were evaluated as significantly negative prognostic factors for overall survival in Cox multivariate models.

## 4. Discussion

Notch signaling drives many cellular processes and identifies as an attractive therapeutic target for uterine cancer as it is essential for the endometrial change processes [22]. Therefore, a therapeutic approach that targets specific receptors or ligands of the Notch signaling pathway that stimulate tumour cell differentiation and progression may provide more effective treatment of uterine cancer patients [23].

As expected, the analysis of our study data showed that the survival rate of patients with uterine cancer is affected by the stage of the disease, histologic type of tumour, tumour differentiation grade, and lymphovascular invasion. In our study, the myometrial invasion was not a statistically significant factor influencing patient survival.

The examination of the influence of Notch signaling pathway components on patient survival revealed a statistically significant association between the uterine cancer survival rate and *NOTCH2–4*, *JAG2*, and *HES1* gene expression, as cases with the higher expression level of the *NOTCH* gene has a worse prognosis of the disease. There is limited evidence in the literature that the *NOTCH2* receptor is an important factor in predicting the prognosis of the disease in patients with uterine cancer. In a recent publication, it has been reported that high levels of *NOTCH2* expression show a statistically significant correlation with poor overall and disease-free survival time and more advanced ovarian cancer stages. In addition, *NOTCH2*, *NOTCH3*, *DLL3*, *MAML1*, and *ADAM17* were determined as the five most relevant genes in ovarian cancer [24]. Polychronidou et al. [11] showed that higher NOTCH2 protein expression in tumour tissue might be associated with increased relapse and mortality rates of endometrial cancer patients. Also, as compared to low endometrial cancer grades, tumours with grade 3 were more frequently characterized with NOTCH2 and NOTCH3 protein overexpression.

*NOTCH3* is an important member of the *NOTCH* family, which is involved in the development and progression of various cancers by regulating the tumour microenvironment, promoting tumour formation, angiogenesis, migration, and invasion processes [25, 26]. It has been shown that *NOTCH3* is a direct target of tumour-suppressive miR-491-5p and miR-875-5p. The activation of *NOTCH3* is partly associated with the silencing of these two miRNAs. High *NOTCH3* expression significantly correlates with poor survival of gastric cancer patients [27]. Moreover, multivariate Cox regression analysis of the gastric cancer patients' clinical features revealed that *NOTCH3* expression might be used as an independent prognostic factor [28].

The data of our study for the first time demonstrate that the Notch signaling pathway receptor *NOTCH4* may also play an important role in the survival of patients with uterine cancer. Results show that low *NOTCH4* expression levels are associated with longer survival rates. It has been reported that *NOTCH4* is implicated in cancer progression [29]. Williams et al. [10] observed the decreased expression of *NOTCH4* receptor, ligand *JAG1*, and downstream targets *HES1* and *HEY1* in low-grade endometrial cancer, indicating that overall Notch signaling is suppressed in low-grade endometrial cancer. Moreover, *NOTCH4* downregulation is linked to suppressed proliferation and induced apoptosis of Erbb2-negative breast cancer cell lines [30]. Another study also confirmed an association between high levels of *NOTCH4* and aggressive malignant colorectal cancer cell phenotype [31]. Shawber et al. [32] identified that *NOTCH4* is associated with *VEGFR-3* (vascular endothelial growth factor-3), thus promoting cancer lymph node metastases. Yao et al. [33] have demonstrated that cytoplasmic *NOTCH4* expression is related to *Ki67* expression, suggesting that tumour cells with *NOTCH4* overexpression have higher proliferation abilities.

Our study showed that patients with *JAG2* gene expression change less than twofolds are associated with better overall survival rates. Contrariwise, Townsend et al. [9] demonstrated that gene expression analysis between normal and malignant patient samples showed significant elevation of the *JAG2* level in endometrial cancer tissues, but it has no impact on cancer patients survival. *JAG2* is associated with cell growth arresting processes due to its function as a downstream mediator of the Wnt/*β*-catenin signaling pathway [34]. Chen el al. [35] showed that the *NOTCH/JAG2* signaling pathway plays an important role in the regulation of bladder cancer cell proliferation, growth, and invasion processes, thus demonstrating that *JAG2* expression is involved in cancer progression.

Recent studies revealed that a high *HES1* expression level, which was stimulated by aberrant Notch signaling, correlates with increased cell proliferation in pancreatic and colon cancer [36,37]. Meanwhile, downregulation of *HES1* expression is associated with decreased cell proliferation and migration abilities. Gao et al. [38] demonstrate that *HES1* expression is linked to downregulation of *PTEN* and activation of the Akt/GSK3*β* pathway, thus enhancing the invasiveness of cancer cells.

Prognostic classification of endometrial cancer using a molecular approach based on gene panels have a potential clinical usage [39]. Testing of *NOTCH* components signaling in addition to other selective molecular biomarkers [40,41] may help qualify prognosis for women with uterine cancer. Our study suggests that the Notch signaling pathway, including NOTCH receptors, ligands, and target genes are essential to uterine cancer development and prognosis. Aberrant Notch signaling indicates its oncogenic potential in uterine cancer via uncontrolled cellular proliferation and avoidance of apoptosis which are two main features of cancer development.

## 5. Conclusions

Our study shows a significant role of the Notch signaling pathway components in uterine cancer. *NOTCH2*, *NOTCH3*, *NOTCH4*, *JAG2*, and *HES1* may be used as independent and significant prognostic factors for uterine cancer patients.

## Figures and Tables

**Table 1:** Clinical-pathological data of patients participating in the study.

Clinical-pathological characteristics	Number of patients (%)
*FIGO stage*	
IA	51 (46.8)
IB	33 (30.3)
II	8 (7.3)
IIIA	1 (0.9)
IIIB	1 (0.9)
IIIC	9 (8.3)
IVB	6 (5.5)

*The histologic type of tumour*	
Endometrioid adenocarcinoma	90 (82.6)
Serous adenocarcinoma	9 (8.3)
Carcinosarcoma	10 (9.2)

*Tumour differentiation grade*	
G1	38 (34.9)
G2	38 (34.9)
G3	28 (25.7)
Undetermined grade	5 (4.6)

*Metastasis to regional lymph nodes*	
Yes	11 (10.1)
No	98 (89.9)

*Lymphovascular invasion*	
Yes	18 (16.5)
No	91 (83.5)

*Myometrial invasion*	
**<** 1/2	51 (46.8)
≥1/2	58 (53.2)

*Menopausal status*	
Premenopausal	8 (7.3)
Postmenopausal	101 (92.7)

*Body mass index*	
18.5–24.9	9 (8.3)
25.0–29.9	21 (19.3)
≥30	79 (72.5)

**Table 2:** Results of Cox multivariate models (adjustments of each gene expression to age and clinical-pathological characteristics) for overall survival of patients with uterine cancer.

Gene	*p* value	Hazard ratio	95.0% CI^*∗*^ for hazard ratio
Fold change of *NOTCH2* expression			
mRNA decrease over 4 folds (reference group)	—	1	—
mRNA increase or decrease up to 4 folds	**0.003**	3.4	1.5–7.9

Fold change of *NOTCH3* expression			
mRNA decrease over 2 folds (reference group)	—	1	—
mRNA increase or decrease up to 2 folds	**0.044**	2.3	1.0–5.1

Fold change of *NOTCH4* expression			
mRNA decrease more than 1 fold (reference group)	—	1	—
mRNA increase more than 1 fold	**0.013**	3.0	1.3–7.3

*Fold change of JAG2 expression*			
mRNA increase less than 2 folds and decrease (reference group)	—	1	—
mRNA increase more than 2 folds	**0.009**	3.4	1.4–8.5

*Fold change of HES1 expression*			
mRNA increase less than 2 folds and decrease (reference group)	—	1	—
mRNA increase more than 2 folds	**0.009**	4.6	1.5–14.6

^*∗*^CI is the confidence interval.

## Data Availability

The data are available on request.

## References

[1] Sung H., Ferlay J., Siegel R. L. (2020). Global cancer statistics 2020: globocan estimates of incidence and mortality worldwide for 36 cancers in 185 countries. *A Cancer Journal for Clinicians*.

[2] https://www.nvi.lt/naujausi-duomenys/.

[3] Henley S. J., Miller J. W., Dowling N. F., Benard V. B., Richardson L. C. (2018). Uterine cancer incidence and mortality - United States, 1999-2016. *MMWR. Morbidity and Mortality Weekly Report*.

[4] Jayawickcrama W. I. u., Abeysena C. (2019). Risk factors for endometrial carcinoma among postmenopausal women in Sri Lanka: a case control study. *BMC Public Health*.

[5] Mori Y., Yamawaki K., Ishiguro T. (2019). ALDH-dependent glycolytic activation mediates stemness and paclitaxel resistance in patient-derived spheroid models of uterine endometrial cancer. *Stem Cell Reports*.

[6] Singh N., Hirschowitz L., Zaino R. (2019). Pathologic prognostic factors in endometrial carcinoma (other than tumor type and grade). *International Journal of Gynecological Pathology*.

[7] Creasman W., Odicino F., Maisonneuve P. (2006). Carcinoma of the corpus uteri. *International Journal of Gynecology & Obstetrics*.

[8] Malik T. Y., Chishti U., Aziz A. B., Sheikh I. (2016). Comparison of risk factors and survival of type 1 and type II endometrial cancers. *Pakistan Journal of Medical Sciences*.

[9] Townsend M. H., Ence Z. E., Felsted A. M. (2019). Potential new biomarkers for endometrial cancer. *Cancer Cell International*.

[10] Williams E., Villar-Prados A., Bowser J., Broaddus R., Gladden A. B. (2017). Loss of polarity alters proliferation and differentiation in low-grade endometrial cancers by disrupting Notch signaling. *PLoS One*.

[11] Polychronidou G., Kotoula V., Manousou K. (2018). Mismatch repair deficiency and aberrations in the Notch and Hedgehog pathways are of prognostic value in patients with endometrial cancer. *PLoS One*.

[12] Daley-Brown D., Harbuzariu A., Kurian A. A., Oprea-Ilies G., Gonzalez-Perez R. R. (2019). Leptin-induced Notch and IL-1 signaling crosstalk in endometrial adenocarcinoma is associated with invasiveness and chemoresistance. *World Journal of Clinical Oncology*.

[13] Nowell C. S., Radtke F. (2017). Notch as a tumour suppressor. *Nature Reviews Cancer*.

[14] Vinson K. E., George D. C., Fender A. W., Bertrand F. E., Sigounas G. (2016). The Notch pathway in colorectal cancer. *International Journal of Cancer*.

[15] Shang C., Lang B., Meng L.-r. (2018). Blocking NOTCH pathway can enhance the effect of EGFR inhibitor through targeting CD133+ endometrial cancer cells. *Cancer Biology & Therapy*.

[16] Janghorban M., Xin L., Rosen J. M., Zang X. H. F. (2018). Notch signalling as a regulator of the tumor immune response: to target or not to target?. *Frontiers in Immunology*.

[17] Liu L., Zhang L., Zhao S. (2019). Non-canonical notch signaling regulates actin remodeling in cell migration by activating PI3K/AKT/Cdc42 pathway. *Frontiers in Pharmacology*.

[18] Li L., Tang P., Li S. (2017). Notch signaling pathway networks in cancer metastasis: a new target for cancer therapy. *Medical Oncology*.

[19] Jonusiene V., Sasnauskiene A., Lachej N. (2013). Down-regulated expression of Notch signaling molecules in human endometrial cancer. *Medical Oncology*.

[20] Lachej N., Jonušienė V., Mažeikė A. (2019). Changes in the expression of Notch and Wnt signalling molecules in human endometrial cancer. *Acta Medica Lituanica*.

[21] Budczies J., Klauschen F., Sinn B. V. (2012). Cutoff Finder: a comprehensive and straightforward Web application enabling rapid biomarker cutoff optimization. *PLoS One*.

[22] Sasnauskienė A., Jonušienė V., Krikštaponienė A. (2014). NOTCH1, NOTCH3, NOTCH4, and JAG2 protein levels in human endometrial cancer. *Medicina*.

[23] Kumar S., Srivastav R. K., Wilkes D. W. (2014). Estrogen-dependent DLL1-mediated Notch signaling promotes luminal breast cancer. *Estrogen-dependent DLL1-Mediated Notch Signaling Promotes Luminal Breast Cancer*.

[24] Jia D., Underwood J., Xu Q., Xie Q. (2019). NOTCH2/NOTCH3/DLL3/MAML1/ADAM17 signaling network is associated with ovarian cancer. *Oncology Letters*.

[25] Huang Q., Li J., Zheng J., Wei A. (2019). The carcinogenic role of the notch signaling pathway in the development of hepatocellular carcinoma. *Journal of Cancer*.

[26] Zhang X., Shi H., Yao J. (2020). FAM225A facilitates colorectal cancer progression by sponging miR‐613 to regulate NOTCH3. *Cancer Medicine*.

[27] Kang W., Zhang J., Huang T. (2021). NOTCH3, a crucial target of miR-491-5p/miR-875-5p, promotes gastric carcinogenesis by upregulating PHLDB2 expression and activating Akt pathway. *Oncogene*.

[28] Cui Y., Li Q., Li W. (2021). NOTCH3 is a prognostic factor and is correlated with immune tolerance in gastric cancer. *Frontiers in Oncology*.

[29] Sun Y., Lowther W., Kato K. (2005). Notch4 intracellular domain binding to Smad3 and inhibition of the TGF-*β* signaling. *Oncogene*.

[30] Yamaguchi N., Oyama T., Ito E. (2008). NOTCH3 signaling pathway plays crucial roles in the proliferation of ErbB2-negative human breast cancer cells. *Cancer Research*.

[31] Shaik J. P., Alanazi I. O., Pathan A. A. K. (2020). Frequent activation of notch signaling pathway in colorectal cancers and its implication in patient survival outcome. *JAMA Oncology*.

[32] Shawber C. J., Funahashi Y., Francisco E. (2007). Notch alters VEGF responsiveness in human and murine endothelial cells by direct regulation of VEGFR-3 expression. *Journal of Clinical Investigation*.

[33] Yao K., Rizzo P., Rajan P. (2011). Notch-1 and notch-4 receptors as prognostic markers in breast cancer. *International Journal of Surgical Pathology*.

[34] Vaish V., Kim J., Shim M. (2017). Jagged-2 (JAG2) enhances tumorigenicity and chemoresistance of colorectal cancer cells. *Oncotarget*.

[35] Chen Y.-T., Huang C.-R., Chang C.-L. (2020). Jagged2 progressively increased expression from Stage I to III of Bladder Cancer and Melatonin-mediated downregulation of Notch/Jagged2 suppresses the Bladder Tumorigenesis via inhibiting PI3K/AKT/mTOR/MMPs signaling. *International Journal of Biological Sciences*.

[36] Abel E. V., Kim E. J., Wu J. (2014). The Notch pathway is important in maintaining the cancer stem cell population in pancreatic cancer. *PLoS One*.

[37] Weng M., Tsao P., Lin H. (2015). Hes1 increases the invasion ability of colorectal cancer cells via the STAT3-MMP14 pathway. *PLoS One*.

[38] Gao F., Huang W., Zhang Y. (2015). Hes1 promotes cell proliferation and migration by activating Bmi-1 and PTEN/Akt/GSK3*β* pathway in human colon cancer. *Oncotarget*.

[39] López-Reig R., Fernández-Serra A., Romero I. (2019). Prognostic classification of endometrial cancer using a molecular approach based on a twelve-gene NGS panel. *Scientific Reports*.

[40] Kim S. R., Cloutier B. T., Leung S. (2020). Molecular subtypes of clear cell carcinoma of the endometrium: opportunities for prognostic and predictive stratification. *Gynecologic Oncology*.

[41] Coll-de la Rubia E., Martinez-Garcia E., Dittmar G., Gil-Moreno A., Cabrera S., Colas E. (2020). Prognostic biomarkers in endometrial cancer: a systematic review and meta-analysis. *Journal of Clinical Medicine*.

