# Supplementary material for: Components of NOTCH Signaling for Uterine Cancer Patients' Prognosis
Source: J Oncol. 2022 Jan 30;2022:8199306. doi: 10.1155/2022/8199306 (PMC8818413; doi:10.1155/2022/8199306)
Supplement: Supplementary Materials — Table S1 and Table S2 are provided as separate word files. [file 8199306.f1.zip › 8199306.f1/Supplement table 1.doc]

**Table S1. Analysis of NOTCH2, NOTCH3, NOTCH4 expression according to clinical-pathological characteristics of uterine cancer patients.**

| **Clinical-pathological characteristics** | ***NOTCH2*** | | ***P*-value** | ***NOTCH3*** | | ***P*-value** | ***NOTCH4*** | | ***P*-value** |
| --- | --- | --- | --- | --- | --- | --- | --- | --- | --- |
|  | ***mRNA decrease over 4 folds*** | ***mRNA increase or decrease up to 4 folds*** |  | ***mRNA decrease over 2 folds*** | ***mRNA increase or decrease up to 2 folds*** |  | ***mRNA decrease over 1 fold*** | ***mRNA increase over 1 fold*** |  |
| **Age**  < 60  ≥ 60 | 14  40 | 16  39 | 0.876 | 19  48 | 11  31 | 0.979 | 28  66 | 2  12 | 0.341 |
| **Stage**  I-II  III-IV | 45  9 | 47  8 | 0.967 | 55  12 | 37  5 | 0.569 | 79  15 | 12  2 | 0.617 |
| **Treatment**  Surgery  Postoperative treatment | 23  31 | 26  29 | 0.765 | 29  38 | 20  22 | 0.806 | 40  54 | 8  6 | 0.461 |
| **Histological type**  Endometrioid adenocarcinoma  Carcinosarcoma  Serous adenocarcinoma | 49  3  2 | 41  7  7 | 0.079 | 57  5  5 | 33  5  4 | 0.664 | 78  9  7 | 11  1  2 | 0.674 |
| **Tumour differentiation grade**  G1  G2  G3 | 15  26  12 | 23  12  16 | **0.025** | 19  28  19 | 19  10  9 | 0.086 | 31  36  22 | 6  2  6 | 0.140 |
| **Lymphovascular invasion (LVI)**  **LVI (-)**  **LVI (+)** | 46  8 | 45  10 | 0.829 | 58  9 | 33  9 | 0.407 | 78  16 | 12  2 | 1.000 |
