# Supplementary material for: Components of NOTCH Signaling for Uterine Cancer Patients' Prognosis
Source: J Oncol. 2022 Jan 30;2022:8199306. doi: 10.1155/2022/8199306 (PMC8818413; doi:10.1155/2022/8199306)
Supplement: Supplementary Materials — Table S1 and Table S2 are provided as separate word files. [file 8199306.f1.zip › 8199306.f1/Supplement table 2.doc]

**Table S2.** Analysis of JAG2, DLL1, HES expression according to clinical-pathological characteristics of uterine cancer patients.

| **Clinical-pathological characteristics** | ***JAG2*** | | ***P*-value** | ***DLL1*** | | ***P*-value** | ***HES1*** | | ***P*-value** |
| --- | --- | --- | --- | --- | --- | --- | --- | --- | --- |
|  | ***mRNA increase less than 2 folds and decrease*** | ***mRNA increase over 2 folds*** |  | ***mRNA increase less than 2fold and decrease*** | ***mRNA increase over 2 folds*** |  | ***mRNA increase less than 2fold and decrease*** | ***mRNA increase over 2 folds*** |  |
| **Age**  < 60  ≥ 60 | 29  67 | 1  11 | 0.173 | 27  66 | 2  11 | 0.507 | 28  72 | 2  6 | 1.00 |
| **Stage**  I-II  III-IV | 81  15 | 10  2 | 1.00 | 79  14 | 10  3 | 0.434 | 85  15 | 6  2 | 0.609 |
| **Treatment**  Surgery  Postoperative treatment | 45  51 | 3  9 | 0.220 | 42  51 | 4  9 | 0.384 | 45  55 | 3  5 | 0.730 |
| **Histological type**  Endometrioid adenocarcinoma  Carcinosarcoma  Serous adenocarcinoma | 80  10  6 | 9  0  3 | 0.054 | 78  8  7 | 9  2  2 | 0.431 | 84  10  6 | 5  0  3 | **0.007** |
| **Tumour differentiation grade**  G1  G2  G3 | 33  34  24 | 4  4  4 | 0.878 | 31  35  24 | 4  3  4 | 0.707 | 35  36  24 | 2  2  4 | 0.320 |
| **Lymphovascular invasion (LVI)**  **LVI (-)**  **LVI (+)** | 81  15 | 9  3 | 0.418 | 79  14 | 9  4 | 0.229 | 85  15 | 5  3 | 0.127 |
